# Supplementary material for: Acute renal failure in children. Multicenter prospective cohort study in medium-complexity intensive care units from the Colombian southeast
Source: PLoS One. 2020 Aug 24;15(8):e0235976. doi: 10.1371/journal.pone.0235976 (PMC7446789; doi:10.1371/journal.pone.0235976)
Supplement: Attachment — Submitted filename: Response to Reviewers .docx [file pone.0235976.s003.docx]

June,14 2020

**Professor**

**Emmanuel A Burdmann**

**Academic Editor**

**PLOS ONE**

I would like to acknowledge for the special and learning-based reviews received in our manuscript (PONE-D-20-03806R1) entitled “Acute Renal Failure in Children. Multicenter Prospective Cohort Study in Medium Complexity Intensive Care Units from the Colombian Southeast”.

We tried to solve all comments of the second review and follow all suggestions of the reviewers to improve the quality and transparency of our paper in this second round. We carefully considered all reviewers’ comments and we are submitting a detailed list of responses to each comment with quotes from the text.

We hope that our manuscript can fully meet all the PLOS ONE’s publication criteria.

Thank you for your consideration.

Sincerely,

Jessica M. Forero on behalf of the authors

**Detailed responses to** each comment.

| **General comments** |  |
| --- | --- |
| 1. If the authors have adequately addressed your comments raised in a previous round of review and you feel that this manuscript is now acceptable for publication, you may indicate that here to bypass the “Comments to the Author” section, enter your conflict of interest statement in the “Confidential to Editor” section, and submit your "Accept" recommendation.  Reviewer #1: All comments have been addressed  Reviewer #2: All comments have been addressed | Thank you for the comment. |
| 2. Is the manuscript technically sound, and do the data support the conclusions?  The manuscript must describe a technically sound piece of scientific research with data that supports the conclusions. Experiments must have been conducted rigorously, with appropriate controls, replication, and sample sizes. The conclusions must be drawn appropriately based on the data presented  Reviewer #1: Yes  Reviewer #2: Yes | Thank you for the comment. |
| 3. Has the statistical analysis been performed appropriately and rigorously?  Reviewer #1: Yes  Reviewer #2: Yes | Thank you for the comment. |
| 4. Have the authors made all data underlying the findings in their manuscript fully available?  The [PLOS Data policy](http://www.plosone.org/static/policies.action#sharing) requires authors to make all data underlying the findings described in their manuscript fully available without restriction, with rare exception (please refer to the Data Availability Statement in the manuscript PDF file).  Reviewer #1: Yes  Reviewer #2: Yes | Thank you for the comment. |
| 5. Is the manuscript presented in an intelligible fashion and written in standard English?  PLOS ONE does not copyedit accepted manuscripts, so the language in submitted articles must be clear, correct, and unambiguous. Any typographical or grammatical errors should be corrected at revision, so please note any specific errors here.  Reviewer #1: Yes  Reviewer #2: Yes | Thank you for the comment. |

| **Reviewer #1** |  |
| --- | --- |
| The revised manuscript shows improvements in relation to the previous one: Title, abstract and introduction are clear | Done. Thank you for the comment. |
| Methodology: the term creatinine serum has to be substituted by serum creatinine | Done. The term was replaced in three situations in the methodology section. |
| Results: where it says " No significant association was noted for gender, age, primary diagnosis, and nutritional status with the presence of severe AKI (Table 2" it should say No significant association was noted for gender, age, primary diagnosis, and nutritional status with the presence of severe AKI (Table 1) | Done. Corrected. |
| Table 2 is confusing as it classifies the patients into mild/moderate and severe AKI and all the p values are non-significant, but the text follows with a paragraph where it is clear that the significant differences were mainly between KDIGO 1 and 3 patients, so table 2 has to be modified and present data classified according to KDIGO status 1, 2 and 3, incorporating all the significant p values shown in the following paragraphs. | Done. As we stated in our methods section Table 1 presents results for mild/moderate KDIGO (st 1) versus severe AKI (st 2-3) measured at admission. Following the reviewer's recommendation, we added two columns to present independently KDIGO stages 1, 2, and 3 and adjusted the next paragraph of the result section called “Association between severity of Acute Kidney Injury on admission to PICU and mortality”.  However, the following paragraphs present details regarding outcomes about the “maximum KDIGO stage reached during the PICU stay”, and not only at admission. To clarify this difference, we adjusted the titles very clear to each section and also we added the Word “maximum KDIGO stage” to each result (highlighted).   - Table 2 title: Outcomes in patients with AKI diagnosis stratified by KDIGO stage, mild/moderate AKI and severe AKI on admission. - Paragraph 1 subtitle: Association between severity of Acute Kidney Injury on admission to PICU and mortality. - Paragraph 2 subtitle: Maximum Stage of Acute Kidney Injury during PICU stay and Outcomes. |
| Discussion is much improved and shows the importance of the presented data to Latin America The text still needs an English language revision. | Done. Thank you for the comment.  Finally, the manuscript was revised again in detail to enhance their language style and grammar. |
